# Alder Hey’s heart of gold: charity, cardiac surgery and the distortion of paediatric provision in a nationalised health service, 1948–91

**DOI:** 10.1136/medhum-2024-013133

**Published:** 2025-03-23

**Authors:** Michael Lambert

**Affiliations:** 1 Medical School, Lancaster University, Lancaster, Lancashire, UK

**Keywords:** health policy, cardiology, paediatrics, medical education, history

## Abstract

Liverpool is perceived as exceptional, a city apart from the nation, and its health services are no different. Alder Hey, the city’s children’s hospital, reaffirms this perspective. Its name is inseparable from the scandal surrounding the unlawful removal and retention of thousands of organs, mostly hearts, from children for research purposes over decades culminating in the 2001 Redfern report. This paper contextualises these events by reconstructing how paediatric cardiac surgery, as an emerging subspecialty, disproportionately shaped the development of children's hospital services for the city and its region. Such cumulative, compounding impacts are invisible in the historiography, focused on national trends and high politics. Recognising but eschewing established policy narratives, the paper follows enduring tensions between teaching and research, service and specialty, centre and periphery, managers and clinicians and patients and professional prestige, which remained unresolved from the inception of the National Health Service (NHS) in 1948 to the Royal Liverpool Children’s Hospital becoming a self-governing trust in 1991. Using archival documents to reconstruct complex organisational and clinical decision-making within the shifting architecture of the NHS at national, regional and local levels over time, this paper shows how children’s hospital services in Liverpool were distorted by another heart. One of gold: charity. Public appeals and philanthropic support for paediatric provision outweighed competing claims for compassionate giving in Liverpool. The availability of alternative funding to develop highly specialised paediatric cardiac surgery, and to lever further statutory resources at the expense of competing specialties, impacted the shape of nationalised health services in the city and its wider region. Ultimately, the paper demonstrates how children’s care, clinicians and charity confounded efforts to organise universal, nationalised health services.

## Introduction

 Finding himself in a ‘dirty, sooty city’ during a lucid dream, psychoanalyst Jung Carl (1963, 223) was famously pulled towards an island of light amid darkness. He declared the place he encountered—Liverpool—to be the ‘pool of life’. In this same light, the city has often been perceived as ‘exceptional’: as a global imperial maritime hub, through the social and cultural character of its people, and as a city apart from recognised national historical narratives (Belchem 2006; Belchem 2007; Haggerty, Webster, and White 2017; Lane 1997). Within this characterisation, Liverpool’s hospitals and health services are no different. Alder Hey, the city’s children’s hospital, reaffirms this perspective. More than 25 years since the scandal first emerged, the hospital’s name is inseparable from the removal and retention of thousands of children’s tissue and organs, mostly hearts, taken without parental knowledge or consent for research (Tomasini 2017, 88–95).

The subsequent Redfern (2001) report laid responsibility for the scandal primarily at the door of an individual aberrant individual pathologist. The wider structures and health systems in which the consultant pathologist worked simply provided the canvas on which the drama unfolded. Questions of accountability and responsibility for senior managers also demanded scalps, including the former Dean of Medicine at the University of Liverpool and the Chief Executive of the Trust. This scandal, combined with another encompassing Bristol, led to national reform with the 2004 Human Tissue Act, which created the Human Tissue Authority, imposing tighter regulation over consent for the removal, storage, use and disposal of human bodies for medical research (Kennedy 2001; Liddell and Hall 2005). Understanding the development of paediatric cardiac surgery, and its consequences for other paediatric provision, is inseparable from the shadow cast by the scandal and the city’s exceptionalism.

Although indelibly associated with Alder Hey, paediatric cardiac surgical operations and postoperative pathology occurred mostly at the Royal Liverpool Children’s Hospital (RLCH) until 1991 when reforms and rationalisation led to its closure. This spatial separation stemmed from historic organisational differences, when the two sites were managed separately. These decisions were the product of accumulated path dependency rooted in historic local health policy decision-making. They produced what Caroline Tuohy (1999: 6) describes as ‘accidental logics’, whereby opportunities and constraints for clinical, managerial and political actors cumulatively and iteratively determine the dynamics of health systems. However, examining these at the level of individual policies, specific reforms or spheres of activities overlooks the wider, ongoing connections such impacts have within local health economies (Tuohy 1999, 261). The inquisitorial nature of the Redfern (2001) report meant the layered contexts in which paediatric cardiac surgery emerged, developed and expanded were marginalised in pursuit of individual culpability. Others have similarly articulated the compounding inheritance of health service organisation as crucial. The late historian John Pickstone (2007) imagined these as ‘contested cumulations’ layered over time, while Lorelei Jones (2018) positions them as ‘sedimented governance’. This paper explores these contested cumulations and their accidental logics in propelling paediatric cardiac surgery as an influential subspecialty. One which disproportionately shaped the development of paediatric health provision in Liverpool and its region.

Constructing a granular case study affords primacy to place. It allows us to examine how competing national policies and priorities—often developed and implemented separately—conflict, interact and are contested within local health economies. This construction compels us to rethink our ideological understanding of the National Health Service (NHS) as a universal, rational and equitably distributed model of healthcare rooted in social democratic aspirations designed to meet patient need regardless of the ability to pay (Seaton 2023; Stewart 2023). At a practical level, focusing on these national parameters and the ‘court politics’ of central government (Ham 2009, 202) creates a disconnect with day-to-day decision-making. These existed outside neatly defined spheres of policy activity and structural reform common to standard narratives of the NHS (Ham 2009; Hardman 2024; Klein 2013; Rivett 1998; Timmins 2017; Webster 2002). These position power as the product of rationality and planning implemented from above within a national model (Stewart 2014, 63), with places understood as problematic because of failures to adhere to central demands.

However, case study approaches that foreground place and the negotiation, contestation and mediation of national policy dynamics in the NHS have been limited (Ham 1981; Lambert 2024a; Mohan 2002). This, despite Martin Gorsky (2008, 453) arguing in his historiographical survey over 15 years ago that “much remains to be learned of how policy translated into practice” through local and regional studies. Paediatric cardiac surgery in the Liverpool region demonstrates the limitations and shortcomings of top-down approaches. Not least because from 1948 until the closure of the RLCH in 1991, policies governing the rise, recognition and regulation of paediatric cardiac surgery were not subject to singular control. They straddled the parallel hierarchies of lay and medical administration of the NHS at the centre (Sheard 2010) and responded to local clinical technical and technological developments. In short, within such a complex environment, access to independent funding such as charity, research or other benefactions and their disproportionate concomitant clinical influence, confounded the equitable delivery of universal, nationalised health services.

A similar position exists in relation to grasping the mixed economy of healthcare after nationalisation in 1948. This acknowledges continuity rather than radical political change (Stewart 2019). Studies have mapped changes in national charitable fundraising from a politically unpalatable inheritance of voluntary hospitals to ideologically desirable with the shift from social democracy to neoliberalism around 1979 (Harris and Cresswell 2024; Stewart, Cresswell, and Möller 2024). Enduring inequalities between places and specialties over their capacity to raise and use non-exchequer funds have also been explored (Abnett, Bowles, and Mohan 2023; Bowles, Clifford, and Mohan 2023; Mohan and Clifford 2024). Work on individual charities has reinforced disease specificity and clinical specialisation in our understanding, being teleological and celebratory in their narration of national discoveries and improvements in treatment (Austoker 1988; Matthews 1990). Local historical studies have focused almost exclusively on hospital Leagues of Friends (Millward 2023; Piggott 2022) rather than the place of charity within the wider local health economy.

In terms of the impact of charity on the policy and ideological objectives of the NHS, a body of literature emerged during the late 1980s. This criticised the impact of charity as a ‘planner’s nightmare’, undermining principles of equalisation and equity (Lattimer 1996, 35), reflecting self-interest among clinicians controlling ‘pots’ of discrete funding for their purposes (Williams 1989, 96) and serving to ‘distort health service planning’ through expenditure on ‘glamorous specialties and technologies’ which burdened limited local budgets (Griffith *et al* 1987, 46). More recently, Mohan and Gorsky (2001, 115) have argued that ‘charitable donations may become an obstacle to the rational reconfiguration of service’. This is evident in two well-known cases. Great Ormond Street Hospital (GOSH) established a ‘wishing well appeal’ in 1987 to subvert plans to close the congested hospital site and rationalise paediatric provision within central London (Arnold-Forster and Gorsky 2024). The heart transplant programme, however, presents the most relevant example. British participation in the international race for a successful transplant programme was suspended by the Department of Health and Social Security (DHSS) in 1973 owing to unpalatably high mortality impacting public support for organ donation. Terence English, subsequently knighted and President of the Royal College of Surgeons, subverted the moratorium in 1979 by obtaining charitable funding from ‘local millionaire David Robinson’ and the British Heart Foundation. Once the operations had proven successful, he received a ‘gentle backhander’ from the DHSS before the ban was formally lifted. The programme was subsequently approved with expensive earmarked central funding in specialist supraregional units (English 2011, 89–115 English in Tansey and Reynolds 1999, 41). Undoubtedly, like GOSH in London, the location of English’s unit at Papworth Hospital in Cambridge benefited from prestigious teaching and research links and contributed to the hospital becoming a self-governing NHS trust in 1992 (Pugh 2015). Both cases are well-known, though, because they centre on prestigious institutions and high-profile successes, unlike Alder Hey and Liverpool.

The specialty example of paediatric cardiac surgery presents further problems of definition. These are central to reconstructing Liverpool’s local health economy. Child health as a branch of medicine was a mainstay of local authority public health services with professional recognition from 1928 (Deborah 1987; Forfar John *et al* 1989). These were particularly prominent in Liverpool, given the city’s association with poverty and associated maternal and infant mortality (Shoemaker 1991). Paediatric surgery was a ‘comparatively new surgical specialty’, which grew with the NHS, only forming a professional subgroup of the British Paediatric Association (BPA) in 1953 (Davenport and De Coppi 2021, 2). This continued with new dedicated, specialist positions replacing those in general surgery with a special interest in paediatrics (Young 2014, 252). Both general and cardiothoracic surgeons contested this paediatric specialty monopoly (Spitz 2012; Zachary 1976) given prevailing models of subspecialisation. These were achieved through the conquest of organ geography (Cooter 1993), whereby technological, biomedical and professional advancements enabled new parts of the body to be subject to medical and surgical intervention (Weisz 2006). This propelled specialisation in the formative years of the NHS in fields such as neurosurgery, urology, haematology and renal medicine. Specialisation and futher subspecialisation was reliant on professionalisation and recognition to enable resources and clinical consideration within the policy apparatus.

Here, Stevens (1966, 377) has argued that cardiology was a ‘spectacular example’ of this dynamic of clinical organisation, leading to the proliferation of the subspecialty. Rivett (2000, 32) suggests that the growth of cardiology was constrained only ‘by the money, buildings and personnel available’, which were worlds apart between the capital and provincial Britain. This surgical conquest of the heart compelled clinicians to move beyond the ‘heroics of the Edwardian world’ (Lawrence 2020, 756). Expensive high technology and multidisciplinary work enabled thoracic surgeons to shift focus within the thorax from the lungs to the heart (Fleming 1997). This propelled the specialty from being a marginal one associated with the stigma of tuberculosis to a prestigious one. Here, cardiologists and cardiothoracic surgeons combined medical and clinical practice in specialist, dedicated NHS units (Coats 2022). These were influential on, but separate from, paediatric practices (Graham and Taylor 2000). Performing such complex operations on children and babies, especially those with congenital heart defects, relied on new anaesthetic techniques. Pioneered in Liverpool (Mulier *et al* 2021), these fuelled neonatal and paediatric cardiac surgical practice, especially in the city itself (Cleland 1983, 893; Spitz 2012, 31). Despite paediatric cardiac surgery being enabled by new technology and specialty expertise, it was not formally recognised until 1990. This ambiguous position had a significant bearing on clinical organisation and practice, and its impact on other forms of paediatric provision within the local health economy.

In moving the locus of analysis to the structures and system of the local Liverpool health economy, paediatric provision and the place of paediatric cardiac surgery within them, aberrant individual behaviour becomes secondary. Understanding paediatric cardiac surgery in Liverpool beyond the organ retention scandal requires the examination of several contested cumulations producing their own accidental logic. These include: enduring tensions between teaching and research needs within hospital and service development; the relative place of an emergent, expensive cardiac subspecialty against other service and patient needs; ambiguous oversight between local and regional health managers and the University of Liverpool, which stems from central uncertainty for responsibility between medical and lay civil servants given the subspecialty delineation; struggles between managers and clinicians in changing policy contexts of cost containment and growth and of professional attitudes towards patients as simply a source of pathology for professional intervention. Following Faye Alberti’s (2010) cultural conception of the heart, at the centre of these developments in Liverpool’s local health economy and the place of paediatric cardiac surgery is not Alder Hey’s heart of darkness, but one of gold. Its heart of gold was made through the acquisition of independent charitable funds by certain clinicians in a context of competition between emergent specialties, and within them, where funding scarcity rendered it impossible for all ambitions and their associated patient needs to be realised simultaneously.

What follows is a chronological analysis of how Alder Hey’s heart of gold distorted the provision of paediatric patient care within Liverpool’s local health economy from the inception of the NHS in 1948 until 1991. This marks the closure of the RLCH, the establishment of Alder Hey as a self-governing NHS trust and the formalisation of the specialty through professional recognition. The paper is reconstructed using archival materials on the margins of national government, regional health authority (RHA) and local hospital and health service files. This is because paediatric cardiac surgery was not a recognised subspecialty and dedicated policy sphere. The focus is not on the technocratic politics of the ‘core executive’ within government, but on the mundane ‘lower levels’ of departmental decision-making (Lowe 1997, 249). Here, sources are fragmentary, offering glimpses and perspectives of emergent and contested decision-making at different hierarchical, professional and organisational levels at different moments in time (Berridge 2001). They are rooted in place and intensely relational across the local health economy. These sources reveal how and why accidental logics are put in motion and sustained, rather than looking for formal, explicit decisions within structures. This opacity reflects intentional obfuscation by actors, and unintentional concealment in records, minutes and papers by junior functionaries (Ruane 2019, 180). In addition, while the recently declassified papers of the Redfern inquiry are useful in their own right, they also permit otherwise unavailable copies of key correspondence and routine reports—typically destroyed in situ, or lost through reorganisational churn (Allan 1982; Higgs and Melling 1997, 128–9)—to be accessed. Beyond the records of the NHS, material from the University of Liverpool archives, the personal papers of key individuals, specialist charities and patient associations from the city and beyond provide important connections to locate power, influence and networks beyond those contained in discrete organisational records.

Reconstructing decision-making through archival fragments makes a methodological virtue of necessity (Gorsky and Mold 2019) despite being ‘extraordinarily time consuming and data hungry’ (Pollitt 2008, 73). However, it also reflects the long shadow of the Alder Hey scandal. In conducting >100 oral history interviews with senior clinicians, managers, politicians, nurses, non-executives and others as part of associated research on the NHS in Liverpool, I encountered widespread reluctance to speak on the record about the development of paediatric services. Where participants were more forthcoming, the strength of emotional feeling around the scandal significantly shaped shared historical memories (Gabriel 2000, 31–57). This limits the utility of elite oral narratives in reconstructing ‘the operation of process and power through different layers of influence’ beyond records (Berridge 2010, 92). Here, Roy Porter’s (1985) exhortation to foreground the patient and approaches from below also presents methodological obstacles. This is through reliance on organisational records presenting the bureaucratic view from within (Lambert 2024) and the hesitation of the Children’s Heart Association—the contemporary successor of the historic charitable patient association linked to paediatric cardiac surgery at Alder Hey—to engage and contribute.

Moreover, while the case study centres on Liverpool, the question of place is far from straightforward. Paediatric cardiac patients requiring surgery were referred from far beyond the city limits, including Barrow and Westmorland to the north, Macclesfield and Cheshire to the east, and from across North Wales to the west. Physical and organisational geographies do not map neatly onto specialty catchments given the larger patient populations required to justify clinical need. Accordingly, the NHS in Liverpool cannot be understood without reference either to the politics of Wales (Webster 2006) or rivalry with Manchester (Lambert 2024a). The NHS in Liverpool was not determined solely by service and policy logics, and its declining social and economic fortunes—along with the wider region—are also significant (Murden 2006) given their influence on hospital rationalisation and relocation. Space and place in the NHS being far more than lines on a map (Lorne and Lambert 2023).

Given its familiarity, periodisation is broadly structured around recognisable narratives from the history of the NHS through key moments of reform. However, underlying continuities, overlap across moments and changes and the involvement and influence of key individuals and institutions over time afford primacy to place. Centred on close reconstruction of policy narratives and their accidental logics traced through fragmented archival sources, the following chronological analysis focuses on how and why local paediatric cardiac surgery exerted such a disproportionate influence in a universal, nationalised health service in one place over time.

## Legacies and inheritance

Mohan (2002, 15) argues that “*explaining* the evolution of systems of hospitals requires… reconstruct[ing] the opportunities and constraints affecting decisions”. The horizons of providing universal, nationalised health services in Liverpool were narrowed through their one principal constraint: the inheritance of the old hospital systems and their deteriorating physical and clinical capacities shaped by a legacy of division. While common across the NHS, these were exceptionally poor for Liverpool. In 1972, the Chief Medical Officer (CMO)—the most senior medical civil servant and the nation’s doctor (Sheard and Donaldson 2006)—wrote to the Permanent Secretary of the DHSS—the most senior lay civil servant responsible for health—to say of Liverpool that (apart from South Wales) “I do not know of another part of the country where everything was bad with quite the same monotony at the beginning of the health service” (Godber 1972). While the CMO is regarded as an ‘unimpeachable source’ by the official historian of the NHS (Webster 1986, 10), others have similarly affirmed the city’s exceptionalism. Lacking ‘a single first-class hospital’ (Ministry of Health 1961), Liverpool ‘found it difficult to break out of the inherited mould of the past’ (Logan Robert *et al* 1971, 1), with constant claims for additional funding or special consideration owing to its exceptionally poor inheritance. Such claims were typically acknowledged *and* granted (Mohan 2002, 125). What had made the city such a problem?

Liverpool’s exceptional inheritance was driven by social and economic inequalities, which produced two contradictory tendencies. On the one hand, the city’s prosperity unleashed a philanthropic boom (Simey 1992). The voluntary hospital movement of the 19th century relied on individual benefaction to endow hospital buildings and beds to care for the poor, with patients admitted through the clinical interest they presented or the intervention of governors affording a human touch. Affluence meant Liverpool’s voluntary hospitals proliferated, spanning the spectrum of specialties. Opened in 1848 to cater solely for children, the RLCH moved twice before securing its lasting premises in the city centre on Myrtle Street in 1866. A country branch was opened in Heswall on the suburban Wirral peninsula in 1899 for rehabilitation. This decision was taken due to the unconducive, polluted atmosphere of the city hospital (Macalister 1930). Yet by this time, there was already a hospital for children since 1869 in nearby Birkenhead, and a new open-air one established in 1912 (Ireland 1938, 57–59). Liverpool’s other voluntary hospitals ribboned the congested docklands and city centre, competing with one another for patronage and patients. Comparatively small, with limited space for expansion and few opportunities to benefit from rationalisation or economies of scale, they were the ‘product of haphazard growth’ (Liverpool Hospitals Commission 1935, 8).

The inability of Liverpool’s voluntary hospitals to meet spiralling running costs through charitable donations or invest in modern medicine was common to interwar Britain. The city’s position was compounded by an abundance of buildings and beds, the waning of philanthropic potential and economic decline, which diluted the value of contributory schemes from insured workers. Such was the scale of crisis that the Ministry of Health was—uniquely, at that time—asked by civic dignitaries to intervene (McIntosh 1933). Reluctantly, this prompted the city’s four main general teaching hospitals to merge and begin fundraising for a replacement. This proposed rebuild was to showcase the city’s clinical potential, curb the ‘danger of surrendering to the new municipal system’ (Liverpool Hospitals Commission 1935, 44) and forestall further state intervention (Hinds 1939).

On the other hand, the city’s health needs, and the failure of philanthropy to meet them, enabled the forward march of the state. Liverpool was the first city to appoint a full-time medical officer of health (MOH), although focused on infectious diseases, sanitation and public nuisances rather than hospitals. Local government parsimony, preference for voluntarism over intervention and running battles over financial responsibility between successive MOH and poor law parishes meant that Liverpool’s municipal public health services were behind the direction of travel by 1914 (Sheard 2006). Concurrent responsibilities for maternal and child health, spurred by anxieties over population fitness during the South African and First World Wars, precipitated an expansion of powers and central government backing with the 1918 Maternity and Child Welfare Act (Deborah 1987). This meant the workhouse planned at Alder Hey in the eastern edges of the city in 1910 instead became a children’s institution from 1918 (Claydon 1991, 13). This process of medicalisation intensified with the 1929 Local Government Act, municipalising poor law responsibilities and bringing them within the empire of the MOH, turning workhouses and institutions into hospitals. Frazer William (1945, 5), the city’s MOH, acclaimed how within 15 years “vast improvements had taken place in the staffing and equipment of the transferred hospitals, and the majority had become efficient hospitals”.

Such modernising claims were hard to sustain, given the Ministry of Health (1938) felt that “an adequately organised municipal hospital service is still comparatively in its infancy”. During the interwar years, the city maintained its reputation as one of the worst for infant and childhood mortality (Liverpool Medical Officer of Health 1938, 17, 18). Indeed, the wartime Domesday survey of hospitals conducted by the Ministry of Health and Nuffield Provincial Hospitals Trust anticipating the NHS felt that it was “high time that the organisation and development of Liverpool’s hospital services were comprehensively planned” (McIntosh and Carling 1945, 49).

In short, Liverpool’s health and hospital services were exceptionally poor, riven by competition between small voluntary hospitals and outdated, under-invested municipal provision. Rationalisation had only begun by 1945, requiring external intervention by government to overcome internal rivalries. Within this, paediatric provision was further diffuse in terms of geography within the city and the region, and as a specialty, with most hospitals continuing to take children as patients and few dedicated paediatric consultant appointments. The mixed economy inherited by the NHS in 1948, then, left an exceptionally problematic legacy for planners.

## Conquering geographies of organ and organisation

Planners faced further problems as specialisation, and its conquest of organ geography, did not align with health service organisations. Owing to political compromises to secure professional commitment, the NHS was not one entity but three, divided between general practitioners and primary care, local authority community care and public health and hospitals. The latter were further bifurcated between Boards of Governors (BoGs), which ran teaching hospitals linked with university medical schools required to teach students and undertake research, and Regional Hospitals Boards (RHBs), which oversaw Hospital Management Committees (HMCs) managing groups of hospitals, each of which had their own House Committees. In large cities such as Liverpool, this bifurcation essentially meant elite former voluntary hospitals retained considerable autonomy under the BoG, while former municipal ones became oriented towards routine patient services under HMCs and the RHB. In Liverpool, the city and country branches of the RLCH were brought under the auspices of the BoG, while Alder Hey provided the heart of Liverpool Region Children’s HMC. The children’s hospitals on the Wirral were grouped separately under North Wirral HMC and Birkenhead HMCs respectively, with paediatric provision planned and delivered independently in each. In its local and regional organisation structures, the NHS in Liverpool inherited and perpetuated its problematic legacy.

Specialisation hinged on the conquest of organ geography. This presented problems for paediatrics, which was concerned with this same conquest, but on newborn, growing and developing children rather than adults. Specialisation required securing substantive paediatric appointments rather than generalists with a special interest. Here, the establishment of a Department of Child Health in 1944 by the University of Liverpool supported by the MOH was significant. Norman Capon, already a Lecturer in Diseases of Children since 1935, was given the inaugural chair (Hay 1977), having unusually held honorary positions at both the voluntary RLCH *and* municipal Alder Hey (Hay 1975). Alongside this, the University of Liverpool Vice Chancellor (1943) recognised that combining bed capacity as RLCH and Alder Hey was vital in making ‘a claim for any financial resources’ from central government for a dedicated paediatric chair. Such resources were in short supply after 1945. Mandated physical improvements at RLCH to enhance the quality and quantity of paediatric teaching were perpetually postponed. These stoked fears there was insufficient ‘clinical material’ to justify the continuation of paediatric teaching at the hospital (Hinds 1958). Despite the distance of several miles rather than a few hundred yards, and the separate organisational purposes of BoGs and RHBs in the NHS, teaching and research were increasingly undertaken at Alder Hey where there was more space and beds, with 900 in comparison with 120 at RLCH (Kelly 1981, 419). The future was jeopardised further as the new single-site teaching hospital designed to replace the four ageing ones was planned with all modern specialties, including paediatrics, in mind (Godber 1953). In organising and planning services given the pre-NHS inheritance and limitations of the RLCH, paediatric provision was vulnerable to subsumption by other specialties and pushed physically and organisationally to the periphery by service logics.

Conversely, the geographical frontiers of organ and organisation advanced dramatically for heart surgery. Historically, owing to technical and technological limitations, thoracic surgeons had been confined to operating on the lungs, associated with the stigma of tuberculosis and physically remote in sanatoria away from busy voluntary and teaching hospitals (Bryder 1988, 173–88). Interwar professional recognition rather than simply a special interest, combined with greater wartime work on traumatic casualties, legitimised thoracic surgery (Timmermann 2014, 53–56). This was deepened through the wartime Emergency Medical Service (EMS), which established regional chest centres to deal with anticipated traumatic casualties, including one at Liverpool. Notwithstanding the 1941 May Blitz, such casualties mostly failed to materialise, and the regional chest centre was repurposed to operate on civilian patients by its director, Hugh Morriston Davies (1942). Located in Broadgreen on the city’s eastern edge, it was less than a mile from Alder Hey. Morriston Davies was succeeded by Ronald Edwards, a ‘master surgeon’ whose career epitomised the shift from risky open cavity surgery to routine patient care for cardiac cases (Bickford 1983). The EMS chest centre became the Liverpool Regional Thoracic Surgery Unit with nationalisation in 1948, based in East Liverpool HMC, although funded through a separate regional allocation (Liverpool Regional Hospital Board Medical Division 1955). Complementing surgical developments were medical ones, which—owing to Liverpool’s legacy—had been fragmented in small clinics of special interest rather than in a single centre (Macalister 1936, 75). Lacking relative specialty status, a Regional Cardiac Centre was established in 1954 in Sefton General Hospital, a former municipal hospital in South Liverpool, by E. N. Chamberlain, who had previously worked in two of the city’s voluntary hospital cardiology clinics (Sanderson 1974). This centre of medical expertise, combined with pioneering anaesthetic techniques (Mulier *et al* 2021), enabled thoracic surgeons to transcend organ geography from the chest to the heart. Their shared physical and organisational space at the periphery of the city, and its legacy of hospital problems, helped shape this dramatic advance.

During the first decade of the NHS, Liverpool’s paediatric provision was driven by organisational logics dividing teaching and research from patient care, and those of organ geography which did not map easily onto children as a distinct patient catchment. These logics mapped onto the physical geography of the city, where the congested RLCH was increasingly unfit for purpose, enabling Alder Hey to expand at its expense despite the best efforts of the city’s health service planners in a context of fiscal stringency. Separately, the shift from thoracic to *cardio*thoracic surgery was also occurring in clinical spaces outside the congested and contested centre of the city at nearby Broadgreen, enabled by multidisciplinary working in allied specialties.

## Specialty and spatiality

Central in combining advances in cardiac care with the fate of paediatrics was the appointment of Professor John Duncan Hay as Capon’s successor in 1957. The son of a cardiologist and Professor of Medicine at the University of Liverpool also named John Hay, Hay sought to square the circle of spatiality and specialty, which had beset Capon’s relocation of paediatric provision from RLCH to Alder Hey (Craft 2004). Trained and educated in Liverpool with an interest in congenital heart defects, expertise in cardiac catheterisation and experience of working in peripheral hospitals where clinical frontiers were advancing, Hay’s appointment signalled a momentous change of direction. Hay (1957) forcefully lobbied the BoG to expand the RLCH by 40 beds to co-locate his professorial unit and the Department of Child Health. This reflected his intention to return paediatrics to the organisational and physical centre of Liverpool ‘where it should be’ (Hinds 1961). The BoG, University and RHB agonised about a ‘fundamental decision’, which achieved little beyond delay. This was because any changes in paediatrics rested on the fate of the delayed new teaching hospital—and money (Hinds 1960). Despite securing tacit consent from the Ministry on the ‘pursuance of a policy’ centralising paediatric provision for teaching and research purposes, money was not forthcoming. This did not prevent Hay relocating his paediatric cardiac patients to the RLCH city and country branches (Freeman 1960) where, unofficially, “quite a lot of the general medical beds [were] being used for cardiac work” (Hinds 1961). “The development of specialised units, particularly cardiac surgery”, warned health leaders, “is tending towards a point of unbalance” in paediatrics at the RLCH (Hinds 1961). The balance being between the general teaching requirements of paediatric patients and pathology for students, and the narrow cardiac research agenda of Hay.

Hay’s slow, unofficial unilateral move of paediatric cardiac work to the RLCH did not go unnoticed. It caused problems for the regional concentration of all cardiac surgery at Broadgreen. This was raised in a memorandum to the BoG by the Professor of Surgery, Charles Wells (1962). He was concerned by the additional costs of diffuse specialty expertise. This, despite none of the specialty consultants being able to operate in all their allocated clinical sessions owing to theatre unavailability (Hamilton 2009, 100). Alongside this, new technical and technological expertise expanded cardiac work, particularly open-heart surgery, through the move from hypothermia to heart-lung machines with Ministry endorsement (Senior Administrative Medical Officers 1958). This also impacted the rationalisation of paediatric and cardiothoracic surgical facilities in North Wales, increasingly run from Liverpool’s regional centres and managed through weekly or fortnightly peripheral clinics by Hay and Edwards (Alwyn-Smith 1965). New consultant appointments such as Douglas Hamilton in 1968 and Ray Donnelly in 1975 continued to split surgical sessions between children and adults, and their time between Liverpool and North Wales (Hamilton 2009, 89; Timmermann 2014, 157–8). Moreover, although with their own children’s hospital and regional paediatric surgical centre, ‘Manchester has not developed neonatal cardiac surgical facilities’, with 30% of Liverpool’s paediatric cardiac surgery cases coming from its rival’s region (Shenokilar 1974). Part of the explanation lies in the establishment of a separate specialist neonatal surgical unit at Alder Hey in 1953 under Isabella Forshall and Peter Paul Rickham (Losty 2024). Operating on neonates with a range of other conditions, most notably spina bifida, the unit ‘became the benchmark’ (Spitz 2003, 1408). Although, like Hay’s paediatric cardiac surgical unit at the RLCH, it had an ‘understated, make-do-and-mend’ ethos in terms of buildings, equipment and staff (Claydon 1991, 55).

Hay’s move initially unsettled this balance of specialty and spatiality, much of which hinged on the delayed decision when and where to build a new teaching hospital, and what specialties to include and exclude from the single site. Its presence in the 1962 Hospital Plan provided some stability to Liverpool’s longstanding hospital problems. This was soon undermined by clinicians frustrated by years of delays and concerned at the relative decline of their own specialty. At Broadgreen, fundraising first by Brigadier Sir Philip Toosey—treated there as a heart patient—then by Sir Douglas Crawford—an influential Tory industrialist and medical philanthropist—generated £80 000 for a new intensive care unit (ICU). The ICU increased postoperative capacity for open heart surgery patients (Hamilton 2009, 105). Crawford was also a significant figure within the Cheshire and Merseyside branch of the British Heart Foundation, whose funds readily flowed into Sefton and the Regional Cardiac Centre (Matthews 1990, 208–10).

Hay capitalised this situation in two ways. First, by securing grant and industry funding for staff including external lectureships, support from children’s formula manufacturer Cow & Gate, and small sums to purchase equipment and disposables (Hay 1977). Second, obtaining £160 000 from local investment magnate Hugh Greenwood—under the guise of the new Children’s Research Fund (CRF)—to build a new Institute of Child Health (ICH) at Liverpool. Opened in 1969, the ICH generated high running costs with new specialist research, laboratory, ancillary and other staff, which the CRF offered a further £30 000 to sustain, subject to matched fundraising from alternative sources. Fundraising failures produced a rising deficit, which led to the University of Liverpool agreeing to shoulder ongoing costs to prevent the ICH closing in 1972. This commitment from the university levered a further £75 000 over 4 years from the RLCH endowment fund (Hay 1977). Crucially, the ‘large teaching and research programme’ (Kelly 1981, 420) driven by Hay continued to focus almost exclusively on congenital heart defects and cardiac malformations.

Access to independent funding by clinicians and specialties could not be taken for granted. Moreover, it was not always spent to return specialties to the physical and organisational centre. For example, John S. Fulton, the director of the regional radiotherapy unit, exploited a substantial charitable bequest to relocate services from Liverpool to the Wirral by 1959 after alienating his cconsultant colleagues in the city (Lambert 2022). Rickham also wrestled for years with the HMC and RHB for funding to modernise neonatal facilities at Alder Hey. This included securing funding from medical equipment manufacturers and requesting help from the Ministry. While sympathetic to his case given the poor quality of facilities, existing expertise and evident clinical need (Todd-White 1964), the Ministry were dismissive of another ‘grandiose scheme’ for Liverpool (Todd-White 1965). Rickham, like hospitals outside the jurisdiction of the BoG, lacked prestige and civic networks through which to mobilise fundraising and raise funds. Moreover, his specialty—spina bifida—although equally lifesaving, did not attract the same glamour as so-called ‘blue babies’ born with a hole in the heart. New operating theatres were eventually opened, but only in 1969. Frustrated, Rickham left Liverpool for a university chair at Zurich in 1971. Rickham’s departure reflected how paediatric cardiac surgery steadily distorted other services within the city and region. These were shaped both by specialty and spatiality of adjacent clinical expertise outside its centre, and the politics of the new teaching hospital.

## Reorganisation and rationalisation

A major structural reorganisation swept away the old tripartite NHS in 1974. Combined with the global economic crisis, decades of failed incremental service rationalisation and subspecialty reform, this created profound changes to Liverpool’s local health economy. Nationally, “as cardiac surgery entered the new phase of rapid expansion, the money dried up” (Rivett 2000, 35). This propelled two tendencies. First, a policy of combining surgical—cardiothoracic—and medical—cardiology—specialties together in single units and sites. This was also intended to maximise clinical benefits from multidisciplinary working, improve training opportunities for junior doctors, shorten waiting lists, and lower patient mortality. Second, one of reducing and rationalising the number of units per population given their high costs. This primarily impacted London where dozens of hospitals undertook specialist, skilled cardiac work at low volume and high cost with mixed patient outcomes. Most NHS regions outside the capital only had one or two such units (Department of Health and Social Security 1973). Paediatric cardiac surgery was planned within the specialty needs of adult cardiology and cardiothoracic surgery, rather than paediatrics, with funding and staffing propelled by these national policy logics. The existing five hospitals with clinical expertise, including RLCH in Liverpool, became designated centres. The Department of Health and Social Security (1974) was focused more on capping overall costs than curbing waiting lists and improving poor-quality premises for such cutting-edge subspecialties. This abnegation by the centre placed greater responsibility on regional and local health authorities to meet the additional costs of such services. Particularly as patients came from other regions and districts with their own budgets and needs, paying little towards the costs despite benefiting from the service. Specialty and spatiality, then, did not conveniently map onto the new landscape of reorganisation and rationality.

Liverpool, and its NHS, was markedly different in 1974 from 1948. In 1948, the city had a population of just under 800 000. By 1974, it was under 600 000 and declining rapidly. Although there was considerable suburbanisation, movement to overspill estates and dormitory towns across the Wirral peninsula, the decrease mirrored the city’s economic fortunes. These, in turn, brought further recognisable social and health inequalities. In 1948, the city hosted the BoG across more than a dozen former voluntary hospitals and six of the RHB’s nineteen HMCs. The 1974 reorganisation replaced these with a single Area Health Authority (AHA) and two District Management Teams (DMTs) based on geography: Eastern, and Central and Southern. BoGs were abolished and teaching hospitals lost their cherished independence. Within the city, paediatric provision remained divided. The RLCH and the much-anticipated new teaching hospital which opened in 1978 fell within Central and Southern DMT. Alder Hey and Broadgreen formed part of Eastern DMT. Despite being across the River Mersey on the Wirral peninsula, Heswall remained the country branch of the RLCH and was funded and governed by Liverpool AHA (T, Teaching), while Birkenhead Children’s Hospital came under the new Northern District of Wirral AHA. Compounding the economic position was the Resource Allocation Working Party (RAWP), a national funding system introduced in 1976. This reduced resource inequalities between NHS regions by basing funding allocations on population need. Owing to its historic legacy and inheritance, the failure of managers to plan comprehensively and decisively, its declining population and proliferation of small specialty units, Liverpool’s health services continued to pose an exceptional problem aggravated by cumulative crises (Logan Robert *et al* 1971).

Countering the continued challenge of rationalisation during the 1970s further distorted the provision of paediatric services and the influence of cardiac surgery. The first overlapped with the 1974 reorganisation and the issue of funding. Expanding capacity at Manchester’s regional cardiothoracic centre at Wythenshawe was used by the DHSS to justify suspending paediatric cardiac surgical growth in Liverpool. They anticipated that patients and procedures from the region who previously travelled to Liverpool would instead be referred to the new centre at Manchester (Shenokilar 1974). Hay responded by privately lobbying several local Members of Parliament (MPs) to ask strategic questions about the size of the paediatric cardiac waiting lists and the proportion of deaths before surgery. Alongside this was local and national press coverage, featuring comment from both the Association of Children with Heart Disorders and Hay, who was also its President (Shenokilar 1974). This archetypal case of shroud waving brought the desired result, and Manchester’s growth was still granted, but not at Liverpool’s expense.

The second challenge focused on the rationalisation of paediatric provision following recommendations by the Department of Health and Social Security (1978). Liverpool Area Health Authority (Teaching) (1979, 22) pushed for the concentration of all paediatric specialties for Liverpool and the Wirral at Alder Hey. This engineered the managed decline of ageing capital and the incorporation of school and community services—common to national policies (Webster 1996, 488–90). The prospect of leaving an ‘isolated paediatric cardiology unit’ within the RLCH was rejected by Liverpool AHA(T) as ‘totally unacceptable’ on both clinical and cost grounds. Despite trenchant local opposition, Birkenhead Children’s and Heswall Hospitals on the Wirral closed in line with this policy of concentration by 1982. Yet, the RLCH deflected their action stemming from the decision. First, because adjacent paediatric surgical subspecialties were mindful of their own futures and opposed the loss of diagnostic equipment, facilities and staff associated with the funding and prestige of paediatric cardiac surgery. Second, the delayed merger of adult cardiology and cardiothoracic surgery at Broadgreen in 1981 again surfaced questions about specialty concentration and co-location (Ennals 1978). This further catalysed opposition to the Alder Hey move, with leading cardiothoracic surgeon David Hamilton dropping adult sessions to concentrate exclusively on paediatric cases and the appointment of a second, dedicated consultant: Roxanne McKay. This preserved the privileged position of paediatric cardiac surgery, but only at the expense of others.

Tellingly, the delay caused by this renegotiation meant much of the ageing capital and equipment justifying the phased closure of the RLCH was again replaced with cutting-edge models, further forestalling concentration at Alder Hey. In addition, the existing six theatres at the RLCH were deemed insufficient to meet growing demand with new procedures and day case capacity by the mid-1980s, leading to the erection of a seventh and the refurbishment of others. This, despite the continued consensus within Liverpool Health Authority (1986a) for a single-site solution for paediatric provision. £30 000 of the cost came from a specific ‘Help a Heartbeat’ appeal, while £125 000 was taken from the Cardiac Fund, which supported adult and paediatric services(Royal Liverpool Children’s Hospital 1988, 6). Despite the economic logic legitimating cuts to costly paediatric cardiac surgery which would redistribute resources for other forms of paediatric care being self-evident to local, regional and national planners, such rationalisation was untenable. Charitable funding buttressed this rearguard action. Exploitation of the emotive aspects sat on top of sedimented layers of cumulative service investment over the preceding decades which frustrated reorganisation.

## Taking heart

By the early 1980s, there was national and professional recognition that paediatric cardiac surgery was not planned as an adjunct of adult activities. “Cardiac surgery in infancy presents special problems”, a Joint Consultants Committee (1981) working party noted: “The initiative of individual paediatric cardiac surgeons, together with their colleagues, has brought the specialty to its present state”. Yet, in arguing for recognition and appropriate resourcing, they recognised that “there is an inescapable conflict between the virtues of centralisation and considerations of geographical convenience”(Joint Consultants Committee 1981). While they meant across regions given consensus around five specialist centres being needed nationally, the same issue was true within Liverpool, where the Joint Consultants Committee (1981) advocated for physical or organisational proximity between paediatric and adult services. The reason for the meeting in 1981 was money, with the patient catchments of such centres larger than regionally organised funding allocations. This led to the introduction of paediatric cardiac surgery as one of several *supra*regional services, with money top-sliced nationally to secure provision because of this funding dilemma (Richings 1982). Liverpool was singled out for performing the highest ratio of operations outside London, benefitting from research into congenital heart defects (Kernohan *et al* 1982). This accentuated the RLCH as a recognised centre of excellence in the subspecialty (Bridgeman 2002), which could take heart in being insulated from wider funding disputes associated with continuing rationalisation and cost improvement policies being pursued across the Liverpool region.

The latest disputes centred on similar issues of disproportionate cost for other subspecialties, namely that Health Authority (HA) allocations under RAWP were not intended to meet the running costs of regional units. These treated patients beyond their organisational and financial boundaries, and most of them were in Liverpool owing to the historical inheritance and reinforcement of subsequent policy logics (Logan Robert *et al* 1971). A new regional funding formula was only introduced in 1986, providing District Managed Regional Units (DMRUs) such as the Regional Adult Cardiothoracic Unit (RACTU) at Broadgreen, among others, with similar protection (Popplewell 1986). Funding security also provided compensation for DMRUs, which wrestled with the latest reform, introduced as part of the 1983 Griffiths Report. This provided clearer lines of authority and accountability through principles of general management. These flowed from the centre to regions and districts, and applied within NHS organisations between districts and hospitals or other services below them, designed as a unit. Liverpool Health Authority (1986b) agonised about the configuration of these service units based on specialty and spatiality, opting for larger acute units centring on the 1974 division of East and Central and Southern over competitive subspecialties.

Paediatrics narrowly avoiding being subsumed into other organisational forms altogether as general management was introduced locally in 1985. Initially, planners continued to arrange services geographically, and it was put into East which was centred on Broadgreen. As planners moved towards specialty as the basis for unit organisation, it was first put into women’s specialty services as one of three with defined population catchments. Separate specialty recognition for paediatrics only came through trenchant lobbying. This crucial separate status owed much to the intervention of Hay’s replacement as head of the Department of Child Health and later Dean of Medicine: Frank Harris. Harris was not a heart specialist like Hay, who had separately secured support for a Professor of Paediatric Surgery interested in the heart and neonates in 1974: James Lister (Dean and Tam 2004). Instead, Harris pursued nationally recognised and funded cardiac expertise to secure recognition for his broader specialty of paediatrics in a context of immense vulnerability. This included appealing unsuccessfully to the University to replace Lister’s chair on his retirement in 1986 (Raine 1986). More successfully, Harris was able to secure the rebuilding of the old ICH at Alder Hey through further CRF funding in 1986. This victory was achieved in the context of significant retrenchment in higher education which significantly impacted the University of Liverpool (Harrop 1994, 65–6), and anticipation about a single-site future for paediatric provision.

By the end of the 1980s, a small cohort of paediatric cardiac surgeons who had fought to retain their bastion at RLCH – despite the new teaching hospital’s orbit – left, despite the continuing growth of their work, as rationalisation became inevitable (Royal Liverpool Children’s Hospital 1988, 7). The gravity of research and teaching at Alder Hey, a combined medical and surgical unit in adult cardiac care at Broadgreen, and recurrent paediatric rationalisation of the 1970s meant the RLCH struggled to justify its position. It is here that the new consultant pathologist at the centre of the Redfern (2001) inquiry arrived, shortly before the move to new purpose-built theatres at Alder Hey in 1990. However, the year was more significant with the transition to an internal market engendered as part of reforms introducing competition into the NHS. Units were invited to become self-governing NHS trusts providing patient services and competing for patient custom from HAs as purchasers. Owing to the political sympathies of the RHA chairman, Liverpool was in the vanguard of these reforms (Lambert 2024a) with political pressure to secure the most first-wave applications. This included the paediatric unit of the HA, spanning what remained of RLCH and Alder Hey, with a new Chief Executive tasked with securing consultant support (Lambert *et al* 2020, 75). The loss of the old consultant guard at RLCH at this crucial juncture proved pivotal.

Crucially, the application to become an NHS trust made much of two traditions distorting the shape of paediatric provision: cardiac surgery and charity. First, the international prestige of the subspecialty, size of patient population, links with teaching and research and advancing technical and technological developments made a compelling case (Royal Liverpool Children’s Hospital and Community Services 1990, 25). Second, and relatedly, were alternative funding sources. These were of considerable interest to regional and national assessors considering financial independence and competition for patients between hospital providers. Among its sources were the hospital’s historic endowment fund of £4 million, and the prospect of future charitable giving associated with a 75th anniversary appeal (Royal Liverpool Children’s Hospital and Community Services 1990, 78). A further £1.5 million from Ronald McDonald’s Children’s Charities for family accommodation was later secured (Claydon 1991, 132–3). Together, charity and cardiac subspecialisation ensured that the heart of gold which sustained the RLCH was transplanted to Alder Hey as part of the vanguard of first-wave NHS trusts in 1991.

While paediatric cardiac provision was finally subsumed within the new organisational logics of NHS trusts and the Royal Liverpool Children’s Hospital and Community Services as a specialist paediatric provider, it remained at its heart through professional recognition, financial security and enduring relationships with the University. The same dynamics underpinned other small, specialist units and services becoming NHS trusts in Liverpool, including RACTU as part of the same first wave in 1991. However, the underlying accidental logic of independent subspecialty units also undermined the attempts by planners to comprehensively plan services for Liverpool (Harris 1989). Ultimately, sedimented legacies before and throughout the NHS of independent resources, primarily charitable funding controlled by clinicians, continued to confound rationalisation through its ability to distort service priorities and their provision.

## Conclusion

Despite the comparative insignificance of the sums when compared with exchequer spending, charity played a significant role in the NHS because it had the power to subvert planning intentions, which remained opaque, contested and ongoing. This, despite the myriad reports and reviews seeking to rationalise services both before and after 1948. The case of paediatric cardiac surgery poses problems of specialisation and organisation, falling between paediatrics, cardiology and cardiothoracic surgery, a problem mirrored in its status as a sphere of activity below the level of hospital service grouping; whether HMC (1948–74), DMT (1974–82) or HA (1982–91). It points to the limitations of approaching the history of the NHS as a cultural symbol or national institution from the top down, given bottom-up local and regional exigencies persisted before and after the appointed day of 5 July 1948. Charity was also sought and spent well before it received ideological sanction from 1979, serving both supplementary and complementary purposes in relation to existing NHS provision. Clarity on what constituted inappropriate uses of charity or sphere which were the responsibility of the exchequer remained elusive. National funding and policy mechanisms remained too blunt to square the circle between local knowledge, geography and discretion on the one hand, and the demands of national, universal, equitably distributed health services on the other. Local knowledge was plural rather than singular, with charitable funding independent of national allocations enabling clinicians greater control in service and specialty rationalisations over administrators and managers. This distorted and unbalanced their priorities. Moreover, the analytical notion of ‘punctuated equilibrium’ marked by ‘path dependency’ exploited at key ‘windows of opportunity’ does not neatly apply in the case of Liverpool. A stable equilibrium was a façade. Incremental planning and service development represented a contest between, rather than balance of, forces. Local moments of change do not align with national directives and the established narrative of reform, reflecting the agency and networks of key individuals and institutions, particularly the University of Liverpool.

In understanding its capacity to distort service intentions and provision, charity within the NHS needs to be situated as a process rather than an event. Under Sir Terence English at Cambridge, charity had the capacity to subvert a clear central decision made by both lay and medical civil servants regarding the moratorium of the heart transplant programme. What occurred in Liverpool over a 40-year period from 1948 to 1991 was a process rather than a single event. Access to independent funding was used to influence debates, contest decision-making, challenge outcomes and—in some cases—act against agreed decisions and lines of policy, such as Hay’s actions over bed categorisation and allocation. Moreover, the impacts should not just be seen in terms of the subspecialty, but in relation to wider paediatric provision, where long-term running costs picked up by the local health economy impacted other units and spheres of activity, such as closures at Heswall and Birkenhead. The RLCH continued to survive despite the weight of received managerial opinion. These small, often incremental actions should be seen below the explicit purpose of formal records, and the careful analytical reconstruction presented here surfaces the contingent, emergent, contested and complex processes which saw the ascendancy of paediatric cardiac surgery.

Such an approach is not without its limitations, with depth coming at the expense of breadth and lacking comparison. Nevertheless, the paper points to the value of a local, relational case study demonstrating how contested cumulations, and their inexorable accidental logics, shape the realisation of patient care in a nationalised health service. Money, however, changes everything, and an applied understanding of how charity was used to distort, rather than what its endorsement represents about ideals of universalism within the established narrative, allows us to grasp the complex realities of its significance in policy-making, and what this means across place and time.

## Data Availability

Data sharing not applicable as no datasets generated and/or analysed for this study.
